# Modern Developments in Bifunctional Chelator Design for Gallium Radiopharmaceuticals

**DOI:** 10.3390/molecules28010203

**Published:** 2022-12-26

**Authors:** Patrick R. W. J. Davey, Brett M. Paterson

**Affiliations:** 1 School of Chemistry, Monash University, Clayton, VIC 3800, Australia; 2 Monash Biomedical Imaging, Monash University, Clayton, VIC 3800, Australia; 3 Centre for Advanced Imaging, The University of Queensland, St Lucia, QLD 4072, Australia

**Keywords:** gallium, radiopharmaceutical, bifunctional chelator, gallium-68, PET

## Abstract

The positron-emitting radionuclide gallium-68 has become increasingly utilised in both preclinical and clinical settings with positron emission tomography (PET). The synthesis of radiochemically pure gallium-68 radiopharmaceuticals relies on careful consideration of the coordination chemistry. The short half-life of 68 min necessitates rapid quantitative radiolabelling (≤10 min). Desirable radiolabelling conditions include near-neutral pH, ambient temperatures, and low chelator concentrations to achieve the desired apparent molar activity. This review presents a broad overview of the requirements of an efficient bifunctional chelator in relation to the aqueous coordination chemistry of gallium. Developments in bifunctional chelator design and application are then presented and grouped according to eight categories of bifunctional chelator: the macrocyclic chelators DOTA and TACN; the acyclic HBED, pyridinecarboxylates, siderophores, tris(hydroxypyridinones), and DTPA; and the mesocyclic diazepines.

## 1. Introduction

Non-invasive molecular nuclear imaging has become a valuable tool to assist clinicians diagnosing and treating certain diseases. Single photon emission computed tomography (SPECT) is the most routinely used nuclear imaging technique. SPECT utilises radionuclides that emit γ radiation which penetrates the body for external detection [1]. The wide availability of technetium-99m (^99m^Tc) (t_1/2_ = 6.01 h) from ^99^Mo/^99m^Tc generators and the ideal single 140 keV γ emission has made ^99m^Tc coordination complexes for SPECT the most common radiopharmaceuticals used in nuclear medicine [2]. Positron emission tomography (PET) utilises positron emitting radionuclides. As the radionuclide decays, the positrons (β^+^) annihilate with their antiparticles, electrons, up to a few millimetres away from the site of emission, causing two coincident 511 keV γ photons, approximately 180° apart. These photons are converted to a three-dimensional image by a circular ring of detectors [3].

PET offers higher sensitivity and resolution than SPECT, which means that the images generated are often of a higher quality at lower radiotracer concentration (10^−10^ M for PET cf. 10^−6^ M for SPECT) [4,5]. Both techniques are often combined with anatomical imaging techniques such as CT to correlate functional data with anatomical information. The availability of PET has historically been limited because of the need for cyclotron-produced radionuclides, such as fluorine-18 (^18^F) (t_1/2_ = 109.8 min), for use in imaging agents [6]. The growth in the use of PET has been stimulated by the introduction of ^18^F-fluorodeoxyglucose (^18^F-FDG), which is a glucose analogue that allows for the imaging of sites where there is a large cellular energy requirement (e.g., in primary tumour and metastatic cells) [7]. There are several other clinically approved radiopharmaceuticals containing ^18^F, ranging in function from brain imaging to prostate and breast cancers and associated metastases [8].

The high cost of running and maintaining cyclotron facilities, as well as the synthetically challenging formation of covalent C-F bonds for labelling biologically active targeting molecules, has encouraged the use of metallic radionuclides. The formation of coordination complexes using positron emitting radionuclides offers the opportunity for the simple preparation and straightforward incorporation of biologically active molecules to the ligand structure. There are several positron emitting radiometals with suitable half-lives that match the biological residence times of the targeting molecule. The success of the ^99^Mo/^99m^Tc generator for SPECT imaging spurred interest in the development of generator-produced radiometals suitable for PET, such as gallium-68 (^68^Ga), which has since become a practical and successful alternative to cyclotron-produced radionuclides for PET imaging [9]. Compared to cyclotrons, generators do not require special premises with large radiation shielding equipment, specialist personnel for the maintenance of cyclotron equipment, nor a large consumption of energy. 

To apply metallic radionuclides to specific biological applications, it is most often necessary to use chelators that form complexes with high thermodynamic stability and kinetic inertness to avoid transmetallation to competing proteins and hydrolysis of the radiometal. To influence the biodistribution and pharmacokinetics and improve selectivity to disease states in vivo, it is important that the chelators, termed ‘bifunctional chelators’ (BFC), feature a reactive functional group (primary amine, carboxylic acid, isothiocyanate, etc.) that can be tethered to a targeting vector biomolecule, such as a peptide or antibody.

## 2. Aqueous Gallium Chemistry and Gallium Radioisotopes

Gallium is a semi-metallic group 13 element. The chemistry of gallium in physiological systems is dominated by Ga^3+^. Lower oxidation states of gallium are known but are generally unstable in water [10,11,12]. Ga^3+^ is readily hydrolysed at pH > 3 forming insoluble Ga(OH)_3_. Radiolabelling at pH values between 3 and 7 is often difficult due to the low solubility of the Ga(OH)_3_ precipitate (K_sp_ = 7.28 × 10^−36^) [13,14]. The precipitate is amphoteric, forming [Ga(OH)_4_]^−^ and re-dissolving at pH > 7, as demonstrated in the following equilibrium equations:

[Ga(OH_2_)_6_]^3+^ + H_2_O ⇌ [Ga(OH)(OH_2_)_5_]^2+^ + H_3_O^+^

[Ga(OH)(OH_2_)_5_]^2+^ + H_2_O ⇌ [Ga(OH)_2_(OH_2_)_4_]^+^ + H_3_O^+^

[Ga(OH)_2_(OH_2_)_4_]^+^ + H_2_O ⇌ [Ga(OH)_3_]_(s)_ + H_3_O^+^ + 3H_2_O

[Ga(OH_3_)]_(s)_ + OH^−^ ⇌ [Ga(OH)_4_]^−^

Stabilising buffers are used to avoid precipitate formation, with citrate and particularly acetate common choices [7]. With its high charge and small octahedral ionic radius (0.62 Å), Ga^3+^ is classified as a hard Lewis acid [15,16]. Therefore, Ga^3+^ will generally coordinate preferentially to hard Lewis bases, such as those containing nitrogen and oxygen donor atoms. However, sulphur, selenium, and tellurium-containing coordinating systems are known [17,18,19]. Ga^3+^ is generally found to form 4- and 6-coordinate complexes [20,21,22]. The solution and coordination chemistries of Ga^3+^ are somewhat similar to Al^3+^ and In^3+^, and very similar to high-spin Fe^3+^ [7]. Ga^3+^ is expected to follow many of the same chemical pathways as Fe^3+^ in the body, having similar electronegativities (Ga^3+^ = 1.81 Pauling units; Fe^3+^ = 1.83 Pauling units), 4th ionisation potentials (Ga^3+^ = 64 eV; Fe^3+^ = 55 eV), and electron affinities (3rd ionisation potential Ga^3+^ = 30.71 eV; Fe^3+^ = 30.65 eV) [23]. Indeed, the biochemical chelation and protein binding similarities between Ga^3+^ and Fe^3+^ are likely causative of physiological Ga^3+^ activity and uptake. Tissue distribution studies have shown that the majority of administered Ga^3+^ ions bind to iron-transporting proteins, such as transferrin, lactoferrin, and ferritin, with some localising in osteoblasts [23,24]. However, differences in reduction potential (unlike Fe^3+^, Ga^3+^ will not be reduced to Ga^2+^ under physiological conditions) mean that Ga^3+^ does not compete with Fe^2+^ containing molecules, such as heme, in the body [23].

Currently, 30 different isotopes of gallium are known, of which 3 are medically relevant: ^66^Ga, ^67^Ga and ^68^Ga (Table 1) [25]. ^66^Ga and ^67^Ga are cyclotron produced, and ^67^Ga has been used for SPECT imaging. ^66^Ga and ^68^Ga are β^+^-emitters with half-lives of 9.5 h and 67.7 min, respectively. ^68^Ga is by far the most extensively studied for medical purposes, mostly due to the widespread availability of the ^68^Ge/^68^Ga generator, which can be stored on-site in hospitals [26]. In a typical ^68^Ge/^68^Ga generator, ^68^Ge^4+^ is immobilised on a column filled with metal oxide/hydroxide matrices where it spontaneously decays to ^68^Ga^3+^. Most ^68^Ge/^68^Ga generators use acidic eluent (0.1 M HCl) to elute a mixture of [^68^Ga][GaCl_3_], [^68^Ga][GaCl_4_]^−^ or [^68^Ga][Ga(OH_2_)_6_]^3+^ [7]. The long half-life of the ^68^Ge parent isotope (t_1/2_ = 270.95 d) means that a ^68^Ge/^68^Ga generator has a working life of up to one year depending on the initial activity (cf. ~14 d for the ^99^Mo/^99m^Tc generator) [9]. Additionally, ^68^Ga can be produced in a cyclotron in large amounts [27]. This second supply source could potentially be important to mitigate supply issues, as well as allowing for a centralised local site for radiopharmaceutical production. ^68^Ga decays to the stable isotope ^68^Zn via β^+^ emission and electron capture (EC). The 67.7-min half-life allows patients to be scanned in clinics quickly, minimising wait times and radiation exposure to both patients and personnel. ^68^Ga also allows for repetitive examinations due to relatively low radiation exposure. ^68^Ga has a maximum β^+^ energy of 1880 keV, an average β^+^ energy of 890 keV, and an annihilation radiation of 511 keV [26]. Although the higher β^+^ energy means a slightly lower resolution than ^18^F, this provides an adequate level of radioactivity for high quality PET images.

## 3. Bifunctional Chelator Design for Gallium Radiopharmaceuticals

**Figure 1:**
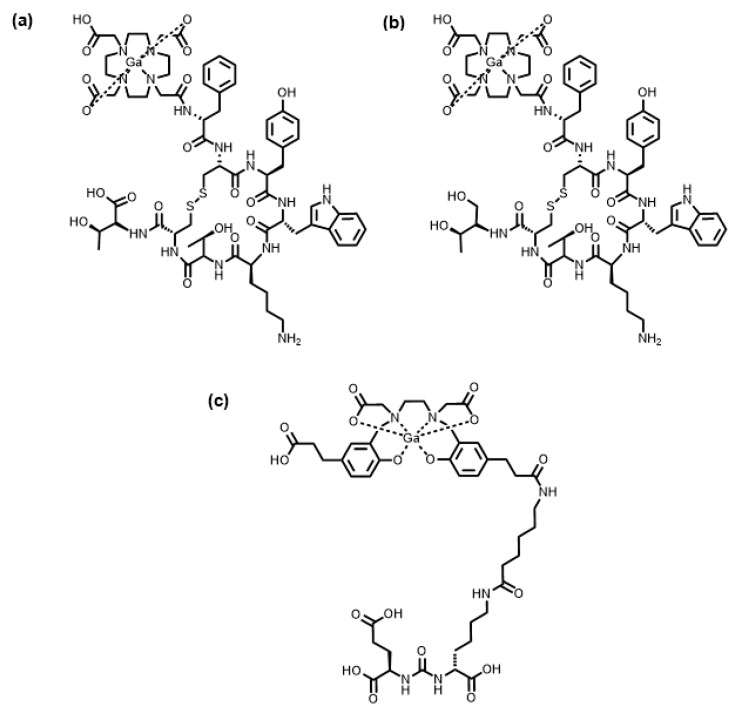
FDA-approved radiopharmaceuticals containing ^68^Ga: (**a**) [^68^Ga]Ga-DOTATATE; (**b**) [^68^Ga]Ga-DOTATOC; (**c**) [^68^Ga]Ga-HBED-CC-PSMA.

There are currently four FDA-approved ^67/68^Ga radiopharmaceuticals in clinical use [8]. Of these, three ^68^Ga radiopharmaceuticals incorporate a BFC tethered to a targeting group via a covalent bond: [^68^Ga]Ga-DOTATATE, [^68^Ga]Ga-DOTATOC and [^68^Ga]Ga-HBED-CC-PSMA (Table 2) [28]. [^68^Ga]Ga-DOTATATE and [^68^Ga]Ga-DOTATOC are both comprised of the 1,4,7,10-tetraazacyclododecane-1,4,7,10-tetraacetic acid (DOTA) macrocycle attached to either the D-Phe^1^-Tyr^3^-octreotate or D-Phe^1^-Tyr^3^-octreotide peptides, respectively, via amide bond formation between a carboxylic acid of the macrocycle and primary amine of the peptide. DOTA has historically been the “workhorse” chelator in metallic radiopharmaceutical development, due to its ability to form complexes with a large number of radiometals, excellent in vivo stability, and commercialisation of various bifunctional derivatives [25]. [^68^Ga]Ga-HBED-CC-PSMA, meanwhile, is comprised of the acyclic chelator, *N*,*N*′-bis [2-hydroxy-5-(carboxyethyl)benzyl]ethylenediamine-*N*,*N*′-diacetic acid (HBED-CC), tethered to a prostate-specific membrane antigen (PSMA)-targeting small molecule motif, glutamate-urea-lysine (Glu-urea-Lys) (Figure 1). HBED-CC found success due to its ability to quantitatively coordinate ^68^Ga^3+^ quickly at room temperature, which was a marked advantage over DOTA [29].

Although [^68^Ga]Ga-DOTATATE, [^68^Ga]Ga-DOTATOC, and [^68^Ga]Ga-HBED-CC-PSMA are used clinically, their syntheses have drawbacks. The radiosynthesis of [^68^Ga]Ga-DOTATATE and [^68^Ga]Ga-DOTATOC requires heating to between 80 and 100 °C to ensure adequate chelation of ^68^Ga^3+^ within the timeframe allowed by the relatively short half-life of 67.7 min [30]. At room temperature, the synthesis of [^68^Ga]Ga-HBED-CC-PSMA produces multiple geometric isomers (in addition to optical isomers) of the [^68^Ga]Ga-HBED-CC complex [31].

Clinical radiosynthesis of the three radiopharmaceuticals takes 5–20 min at pH 3–5 with heating, followed by purification to remove by-products and unreacted ^68^Ga [29]. These conditions add process complexity, limit molar activity (the measured radioactivity per mole of radiopharmaceutical), and the heat and low pH may damage vector biomolecules. The ideal synthesis should be a one-step procedure, matching the simplicity of the long-established kit-based ^99m^Tc radiolabelling protocols [29,32,33,34]. This would enable fast, simple, and reproducible formulations of the radiopharmaceutical in keeping with good manufacturing practice (GMP) standards [35,36]. The design of BFCs that can quantitatively coordinate metallic radioisotopes such as ^68^Ga, with the resulting complexes exhibiting high thermodynamic stability and kinetic inertness in vitro and in vivo, is now considered a mature field [37].

**Figure 2:**
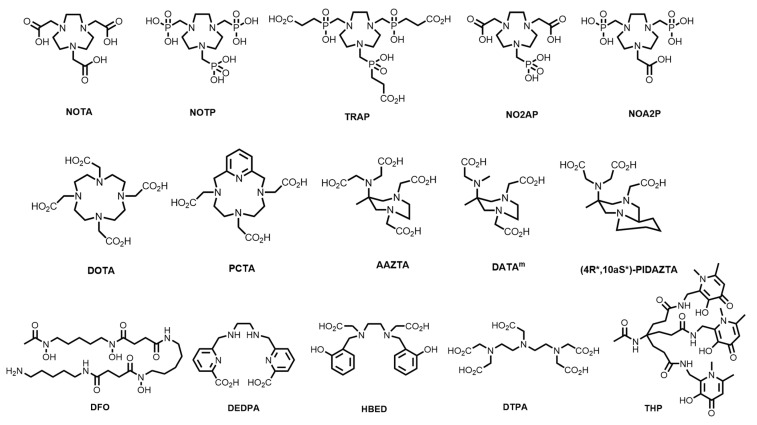
Structures of some common macrocyclic and acyclic chelators for use in ^68^Ga radiopharmaceuticals.

A plethora of bifunctional chelating systems have been developed, with varying stabilities, inertness, radiolabelling conditions, and biological conjugates, to circumvent some of the limitations of both DOTA and HBED-CC in biomolecule radiolabelling [38,39]. These include derivatives of 1,4,7-triazacyclononane (TACN)-based chelators (1,4,7-triazacyclononane-1,4,7-acetic acid, (NOTA), -phosphonic acid (NOTP), and -phosphinic acid (TRAP) and the mixed phosphonate/carboxylate NOA2P and NO2AP) [40]; tetraazamacrocycles (e.g., pyridyl-substituted PCTA) [41]; the acyclic siderophore desferrioxamine-B (DFO) and related siderophores [38]; substituted pyridine carboxylate-based acyclic chelators (e.g., dedpa) [42]; 6-amino-1,4-diazepanes with *N*-substituted pendant arms (e.g., AAZTA, DATAm and PIDAZTA) [43]; *N*-hydroxypyridinones (THP); and other acyclic chelators such as DTPA (Figure 2) [44].

The following sections will discuss the recent developments (2012–2022) of bifunctional chelators for radiolabelling with Ga radioisotopes and are divided by chelator type (beginning with developments in HBED and DOTA bifunctional chelators). A discussion of the acyclic families of chelators (DTPA, siderophores, pyridinecarboxylates, and hydroxypyridinones) is followed by the hybrid acyclic/macrocyclic diazepines, and finally, macrocyclic TACN. A discussion of the ligand design and the coordination chemistry with Ga^3+^, where applicable, is provided as well as progress that has been made to deliver the radiation to specific sites in vivo by tethering the complexes to targeting vectors, such as peptides, antibody fragments, and receptor-specific molecules. In this context, we hope to obtain a comprehensive understanding of the library of bifunctional chelating systems available for Ga radioisotopes, with a view to designing novel systems for specific applications.

## 4. DOTA and Other Tetraazamacrocyclic-Based Bifunctional Chelator Development

**Figure 3:**
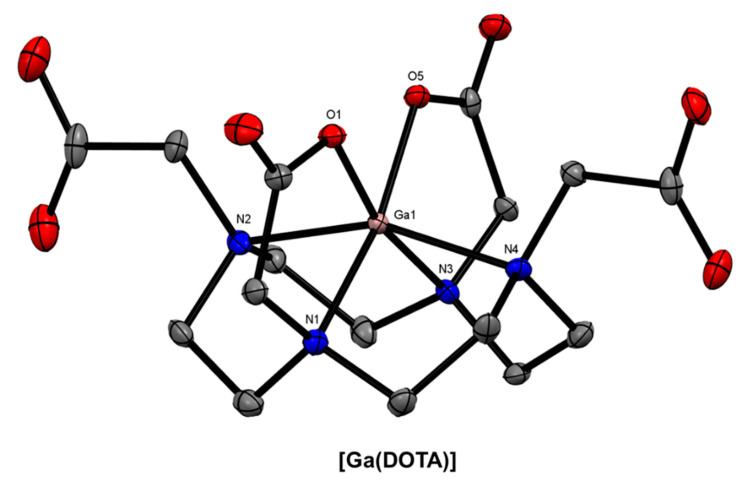
*ORTEP* representation of [Ga(DOTA)] (Cambridge Structural Database Refcode (CSD)-DEVHIW) [46,47]. Ellipsoids are drawn at the 50% probability level. Hydrogen atoms and solvent molecules are omitted for clarity.

The coordination sphere of the Ga^3+^-DOTA complex is a N_4_O_2_ distorted octahedron (Figure 3), and it is well-understood that the large size of the macrocycle is not well-suited for Ga^3+^ complexation (log*K*_1_ = 26.05) [45] compared with more flexible acyclic chelators or smaller macrocycles [25]. As previously mentioned, DOTA and its associated bifunctional derivatives are often used as a motivating factor for researchers to develop better chelating systems for ^68^Ga radiolabelling. However, the fact remains that two of the four ^68^Ga radiopharmaceuticals currently approved by the FDA are based on the DOTA macrocycle. Consequently, effort has been made towards developing DOTA-based BFCs for radiopharmaceutical applications, as well as novel cyclen-based and other tetraazamacrocyclic non-bifunctional ^68^Ga chelators.

**Figure 4:**
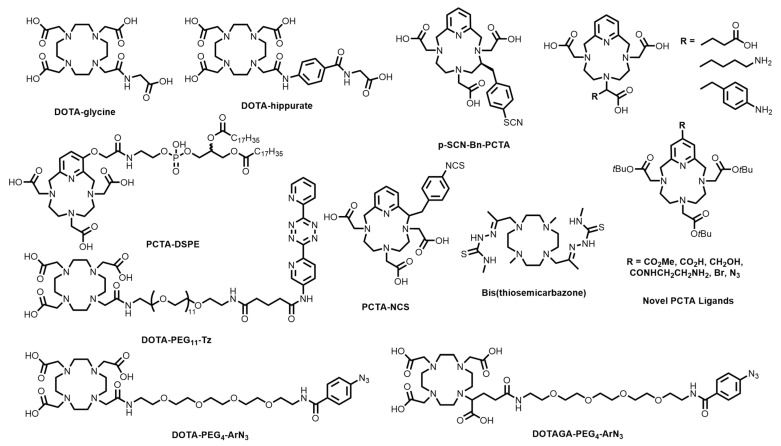
Bifunctional chelators and conjugates based on tetraazamacrocyclic chelators.

Edem and co-workers prepared a poly(ethylene glycol)-tetrazine DOTA conjugate (DOTA-PEG_11_-Tz, Figure 4) that was investigated as a pre-targeting probe for both the ^68^Ga radiolabelling of bone (via conjugation to a trans-cyclooctene (TCO)-derived bone-targeting bisphosphonate, alendronate), as well as the TCO-conjugated antibody, CC49 (which targets the tumour associated glycoprotein 72 antigen on colorectal cancer cells) [48]. The advantage of pre-targeting is that you can heat the complex without damaging the antibody. Additionally, pre-targeting provides an opportunity to use ^68^Ga, with its short half-life, with biomolecules that have long biological half-lives, such as antibodies [49,50,51]. The radiotracers showed target-specific uptake of [^68^Ga][Ga(DOTA-PEG11-Tz)] in the bone (3.7% injected dose/gram (%ID/g) in the knee) in mice pre-treated with TCO-alendronate, as well as tumour-specific uptake (5.8% ID/g) in mice containing LS174 xenografts that were pre-treated with TCO-CC49.

Pathuri and co-workers reported DOTA-hippurate and DOTA-glycine conjugates (Figure 4) with the aim of developing alternative renographic agents to ^99m^Tc-DTPA (used in SPECT) for PET [3]. Both ^68^Ga complexes demonstrated high radiochemical purity (RCP > 98%) at pH 4–5 within 10 min and cleared from circulation primarily through the kidneys (with <0.2% ID/g remaining in the blood at 1 h post-injection (p.i.)). DOTA-hippurate and DOTA-glycine both showed kidney-to-blood %ID/g ratios of ~3:1, which were deemed insufficient for further investigation.

DOTA conjugates containing octreotate [52], antibodies [53], peptides, including arginine-glycine-aspartic acid (RGD) [54,55], and PP-F11 (a target for cholecystokinin-2 receptors, which are overexpressed on small cell lung cancer among others) [56], drug-loading dendrimers [57], neoplastic tissue-targeting porphyrins [58,59], aptamers [60], Fibroblast Activation Protein (FAP) inhibitors [61], nitroimidazoles [62], and siderophores for bacterial imaging [63], have also been prepared and radiolabelled with ^68^Ga in recent years using previously-described amide-bond and thiourea coupling. The N_4_O_3_ DOTA analogue, 3,6,9,15-tetraazabicyclo-[9.3.1]-pentadeca-1(15),11,13-triene-3,6,9-triacetic acid (PCTA), containing a pyridine group in lieu of one of the macrocyclic secondary amines, has shown significant promise with many bifunctional variants reported [64,65,66,67]. Additionally, ^67/68^Ga complexes of PCTA have shown superior human serum stability compared to the DOTA complexes [68]. Bifunctional variants have found success in various studies, including the ^68^Ga radiolabelling of peptides (*p*-NO_2_-Bn-PCTA, Figure 4) [66], RGD radiolabelling (p-SCN-Bn-PCTA, Figure 4) [68], and visualising atherosclerotic plaques (PCTA-DSPE, Figure 4) [69], among other novel PCTA ligands (Figure 4) [68].

A novel bis(thiosemicarbazone)-dimethylcyclen ligand was recently reported and investigated as a chelator for ^68^Ga (Figure 4) [19]. The ligand quantitatively coordinated ^68^Ga^3+^ at 90 °C after 10 min at both pH 3.5 (radiochemical yield (RCY) = 95.1 ± 2.3%) and pH 6 (RCY = 89.0 ± 4.3%), which were similar to results reported for DOTA (pH 3.5 RCY = 95.3 ± 0.9%; pH 6 RCY = 97.2 ± 0.3%) at a chelator concentration of 50 μM. However, at lower temperatures (25 °C and 40 °C), the chelator performed much worse at both pH values. No bifunctional variants of the scaffold have been reported. ^67/68^Ga labelled porphyrins and tetrapyrroles have been reported, however the unfavourable radiolabelling efficiencies may not allow clinical translation [58,70,71,72,73,74].

## 5. HBED-Based Bifunctional Chelator Development

**Figure 5:**
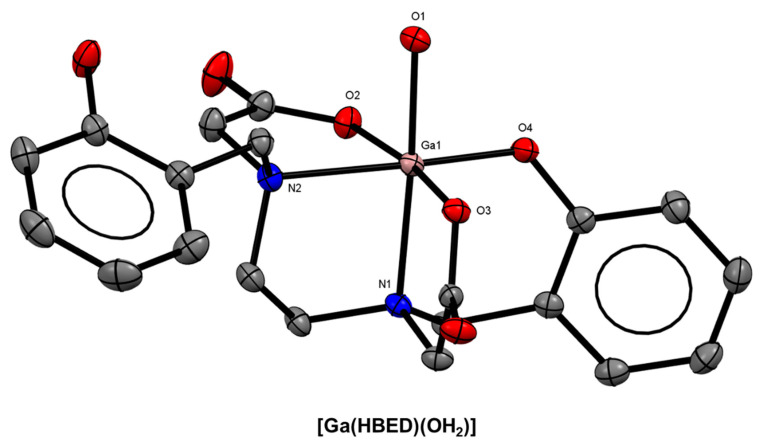
*ORTEP* representation of [Ga(HBED)(OH_2_)] (CSD-PEGSIG) [[38]. Ellipsoids are drawn at the 50% probability level. Hydrogen atoms and solvent molecules are omitted for clarity.

The acyclic chelator, HBED, based on an ethylenediaminetetraacetic acid (EDTA)-type framework with two pendant phenol arms, has several structural characteristics (N and O donor atoms, potential hexadentate coordination environment) that make it an ideal chelator for Ga^3+^. The original chelator synthesis was described in the 1960s [75]. The resulting Ga^3+^ complex is highly thermodynamically stable (log*β*_1_ = 38.51) [76]. An X-ray crystal structure of HBED with Ga^3+^ revealed an N_2_O_4_ octahedral coordination sphere with the N_2_O_3_ pentadentate chelator and an apical water molecule (Figure 5). The development and characterisation of novel bifunctional variants of the dicarboxylate analogue, HBED-CC, has been growing over the past decade since Eder and co-workers’ reported the conjugation of the PSMA-targeting motif, Glu-urea-Lys, to HBED-CC for the imaging of prostate cancer ([^68^Ga]Ga-HBED-CC-PSMA) in 2012 [31,77].

**Figure 6:**
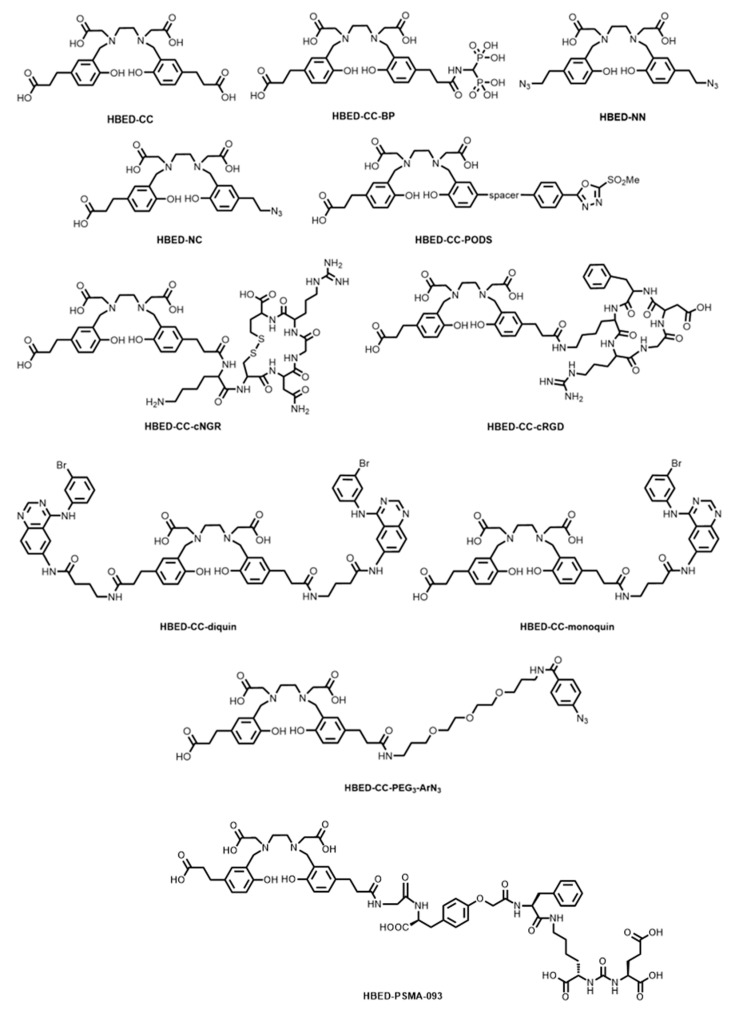
Bifunctional chelators and conjugates based on the HBED platform.

In an effort to improve on the characteristics of Eder’s HBED-CC-PSMA platform, Zha and co-workers introduced an *O*-(carboxymethyl)-L-tyrosine linker group into the structure (termed HBED-PSMA-093, Figure 6), which demonstrated increased cell internalisation (12.5% ID/10^6^ cells at 1 h) compared to HBED-CC-PSMA (7.4% ID/10^6^ cells at 1 h), whilst also demonstrating comparable fast clearance from non-target organs [78]. The development of a robust kit-based synthesis followed, and direct comparison with [^68^Ga]Ga-PSMA-617 (a DOTA analogue) in PET/CT of patients with prostate cancers showed higher tumour uptake, less blood pooling, and reduced bladder accumulation [33,79]. Targeting the HBED-CC chelator for bone imaging was achieved by tethering a bisphosphonate (HBED-CC-BP, Figure 6) [80]. The ^68^Ga complex (termed [^68^Ga]Ga-P15-041) demonstrated excellent in vivo and in vitro stability, and compared favourably to the more widely used tracer, [^18^F]NaF. A kit-based formulation of the complex showed high RCP and radiochemical conversion (RCC) (>90%) within 10 min and required no further purification [81].

Satpati and co-workers reported the conjugation of HBED-CC via amide bond formation to RGD (HBED-CC-cRGD, Figure 6) and asparagine-glycine-arginine (NGR)-containing peptides (HBED-CC-cNGR, Figure 6) for ^68^Ga radiolabelling and PET imaging of tumour vasculature markers, integrin α_v_β_3_ and CD13/aminopeptidase N, respectively. They showed that whilst uptake was similar in HT-1080 human xenografts, the radiolabelled HBED-CC-c(RGD) conjugate displayed higher uptake in B16F10 tumours and higher specificity to α_v_β_3_-positive cells than the NGR conjugate [82].

The conjugation of HBED-CC to monoclonal antibodies (mAbs) and antibody fragments for antibody-based PET imaging (immuno-PET) has also been explored. Fay and co-workers used photoactivatable aryl azides (HBED-CC-PEG_3_-ArN_3_, Figure 6) for the fast (~10 min) conjugation of the HBED-CC chelator to a model anti-c-MET antibody, onartuzumab, via the formation of an azepin group [83]. Klika and co-workers have recently reported a thiol-reactive HBED-CC derivative containing a phenyloxadiazolyl methylsulfone (PODS) group (HBED-CC-PODS, Figure 6) to mitigate the instability of the resulting succinimidyl linkage of the traditional maleimide coupling procedure (the reaction between a thiol of cysteine-containing biomolecules and a maleimide-containing chelator) [84,85].

Analogues of HBED-CC have recently been synthesised for unique targeting applications as well as direct click chemistry reactions. Liolios and co-workers functionalised the propionic acid groups of HBED-CC with the tyrosine kinase (TK) pharmacophore, 4-amino-*N*-(4-((3-bromophenyl)amino)quinazolin-6-yl)-butanamide, to form both the monomeric (HBED-CC-monoquin, Figure 6) and dimeric (HBED-CC-diquin, Figure 6) quinazoline derivatives [86]. TK receptors, including epidermal growth factor receptor (EGFR), are overexpressed in several tumours. Cytotoxicity studies of the ^nat^Ga^3+^ complex in A431 cells (which overexpress EGFR) showed half maximal inhibitory concentration (IC_50_) values in the micromolar range (50–70 μM), the first Ga^3+^-quinazoline derivatives to show this. The ^68^Ga radiolabelled HBED-CC-monoquin complex displayed superior tumour uptake compared to HBED-CC-diquin.

Makarem and co-workers have published the syntheses of two novel azide-containing derivatives, namely the symmetric diazido HBED-NN (Figure 6) and asymmetric monoazido-monocarboxylate HBED-NC (Figure 6), for labelling via the Cu^+^-catalysed azide-alkyne cycloaddition (CuAAC) reaction [87,88]. HBED-NC, in particular, shows significant promise through the potential construction of heterodimeric architectures (i.e., for use in multi-modal imaging procedures such as PET/CT/fluorescence imaging, studies of which were recently reported) [89]. The synthesis of HBED-CC and derivatives has been adapted to solid-phase techniques, which could potentially ease the multistep synthetic protecting group strategies currently employed for the synthesis of HBED-CC derivatives [90].

The approval of [^68^Ga]Ga-HBED-CC-PSMA in December 2020 by the FDA for the PET imaging of prostate cancer has catalysed the developed of novel BFCs based on the HBED scaffold, with renewed interest in the once-overlooked chelating platform [25]. The successful clinical translation of other HBED bioconjugates has yet to be seen. However, it seems likely that the favourable ^68^Ga radiolabelling kinetics of HBED will lead to further radiopharmaceuticals with novel biological targets in future.

## 6. DTPA-Based Bifunctional Chelator Development

**Figure 7:**
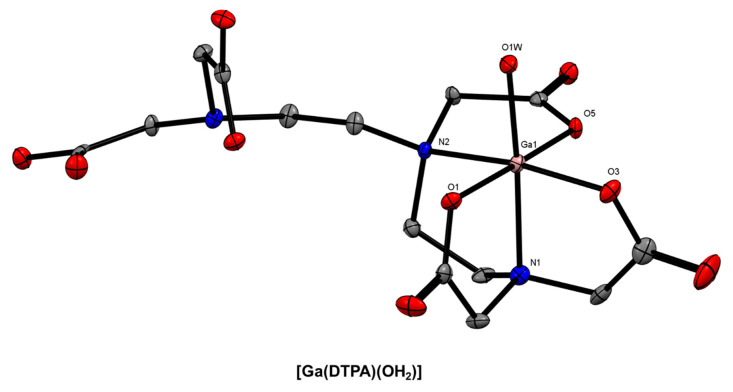
*ORTEP* representation of [Ga(DTPA)(OH_2_)] (CSD-TICDUH01 [93] Ellipsoids are drawn at the 50% probability level. Hydrogen atoms and solvent molecules are omitted for clarity.

Another acyclic chelator that has been investigated as a ^68^Ga^3+^ chelator for radiopharmaceutical development is 2-(bis{2-[bis(carboxymethyl)amino]ethyl}-amino)acetic acid (DTPA) [25,91]. The X-ray crystal structure of the Ga^3+^ complex was reported by Wallin and co-workers and revealed a N_2_O_4_ coordination sphere comprising the 5-coordinate DTPA chelator and an apical water molecule (Figure 7) [92]. This is somewhat surprising given the eight potential donor atoms and is potentially due to the small ionic radius of the Ga^3+^ ion or the conditions of crystallisation. The analogous Fe^3+^ complex, [Fe(DTPA)]^2−^, is 7-coordinate [93,94].

**Figure 8:**
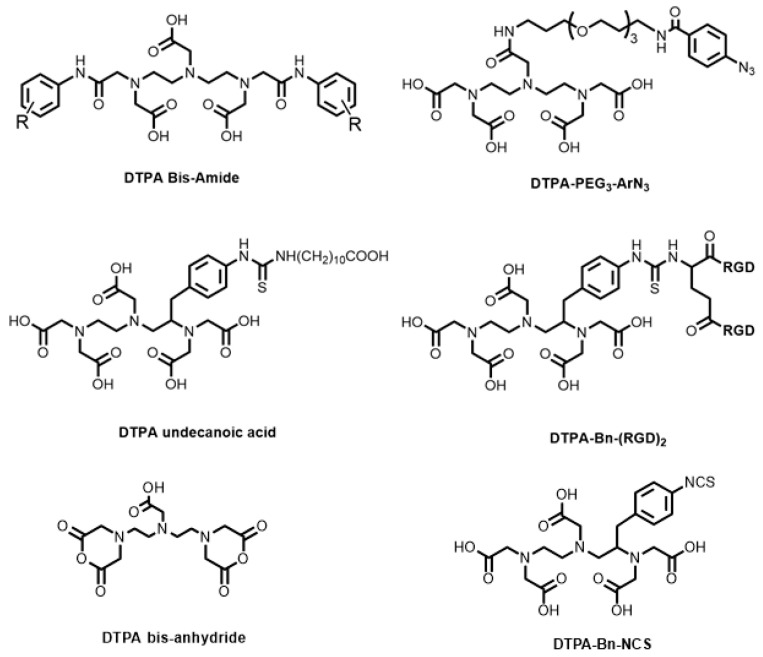
Bifunctional chelators and conjugates based on the DTPA platform.

The synthetic development of DTPA BFCs has involved derivatisation of the carboxylic acids or the incorporation of a functional group to the diethylenetriamine backbone. Bis-amide derivatives (Figure 8) synthesised from DTPA bis-anhydride were shown to form complexes with Ga^3+^, In^3+^, and Lu^3+^ [95]. DTPA bis-anhydride reacts with amine-containing compounds, including biomolecules containing surface lysine residues, to generate the resulting DTPA bis-amide. However, radiolabelling studies with ^68^Ga were not performed. DTPA bis-anhydride was also used to label β-neurotoxins of *Micrurus fulvius* with ^67^Ga to track the biodistribution of the venoms via reaction with the proteins’ surface lysine residues [96].

Gut and Holland utilised similar aryl azide chemistry to the HBED-CC-PEG_3_-ArN_3_ system to produce DTPA-PEG_3_-ArN_3_ (Figure 8) via amide coupling for radiolabelling of a model antibody, trastuzumab, with ^68^Ga [97]. In comparison to HBED-CC-PEG_3_-ArN_3_ (18.5 ± 0.5%, *n* = 2) [83], as well as the NOTA- (15.5 ± 1.5%, *n* = 3) [53], 1,4,7-triazacyclononane-1-glutaric acid-4,7-diacetic acid (NODAGA)- (22.0 ± 3.5%, *n* = 3) [98], DOTA- (12.7 ± 3.2%, *n* = 3, Figure 4) [53], DOTAGA- (11.1 ± 0.2, *n* = 3, Figure 4) [53], and DFO- (67–88%, *n* = 2) analogues [99], DTPA-PEG_3_-ArN_3_ showed the lowest RCC values of the antibody conjugates (3.9 ± 1.0%, *n* = 4), with the precise reasons currently unknown.

Another way to generate DTPA BFCs is via functionalisation of the alkyl backbone. This has the advantage of not interfering with the available donor atoms, allowing for a potential non-coordinating carboxylic acid to be derivatised further. An isothiocyanate (NCS) analogue of DTPA (DTPA-Bn-NCS, Figure 8) was derivatised from the diethylenetriamine backbone. The NCS group reacts with amines under mild conditions to form a thiourea bond.

Jain and co-workers compared the relative stability of various ^68^Ga-labelled bis-RGD peptide conjugates (utilising the DTPA-Bn-NCS, NOTA-Bn-NCS, and DOTA-Bn-NCS BFCs) and found that the DTPA analogue had by far the worst metabolic stability, as well as requiring high temperature radiolabelling conditions to achieve high RCY (along with the DOTA analogue) [100]. The attachment of DTPA-Bn-NCS to a short-chain fatty acid, undecanoic acid (DTPA undecanoic acid, Figure 8), was developed for imaging cardiac metabolic events [101]. However, the biodistribution of the ^68^Ga complex revealed lower myocardial uptake (1.3 ± 0.5% ID/g) compared to the TACN derivatives, NOTA undecanoic acid (3.8 ± 0.6% ID/g), and NODAGA undecanoic acid (3.8 ± 0.6% ID/g).

In recent years, DTPA and derivatives have also been used to decorate iron oxide nanoparticles for ^68^Ga radiolabelling studies, as well as for the molecular imaging of glomeruli using the targeting agent, tilmanocept (using a DTPA-mannosyl-dextran adduct) [102,103]. Taken together, these results seem to suggest that DTPA is not an optimal chelator platform for ^68^Ga, as it often shows relatively poor RCC results compared to other acyclic as well as macrocyclic chelators. With the success of HBED and promising results of other acyclic chelators (vide infra), DTPA has been supplanted.

## 7. Siderophore-Based Bifunctional Chelator Development

**Figure 9:**
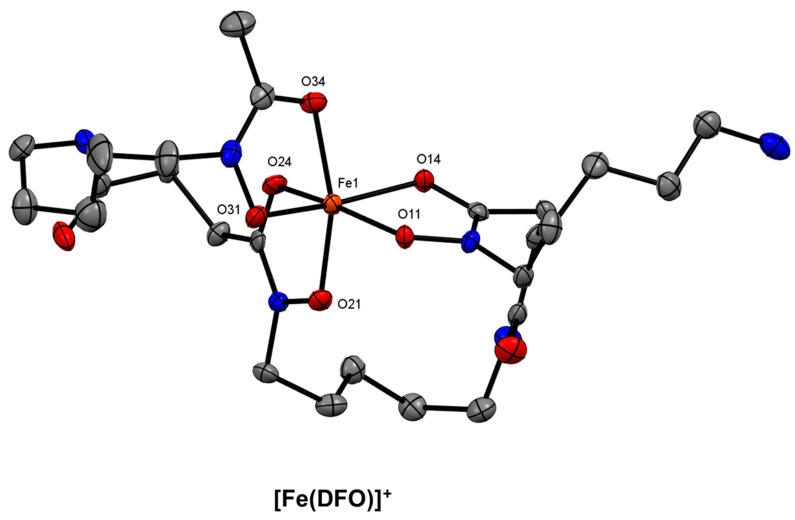
*ORTEP* representation of the [Fe(DFO)]+ cation (CSD-OFUYET) [113]. Ellipsoids are drawn at the 50% probability level. Hydrogen atoms, counterions, and solvent molecules are omitted for clarity.

A promising class of acyclic chelators for ^68^Ga are the siderophores. Siderophores are most well-known as Fe^3+^ sequestration agents used by fungi, bacteria, and plants [104,105]. Due to the chemical similarities between Fe^3+^ and Ga^3+^ (*vide supra*), siderophores have been identified as potential ^68^Ga^3+^ chelating agents. Desferrioxamine-B (DFO) is a bacterial siderophore that was originally characterised in 1958 as a metabolite of *Streptomyces pilosus* [106]. DFO is a linear trihydroxamic acid that chelates Fe^3+^ to form ferrioxamine B ([Fe(DFO)]^+^ or FOB) (Figure 9). DFO was one of the first reported bifunctional siderophores to be radiolabelled with radioisotopes of Ga in high RCY [107]. The DFO-human serum albumin (HSA) conjugate was radiolabelled with ^67^Ga at pH 7–8, achieving a RCY of 99.8 ± 0.3%. Despite the fact that DFO conjugates have been seen to leach radioisotopes of Ga in vivo [108,109,110,111], several bifunctional chelating agents have been synthesised over the past decade based on the DFO scaffold, as well as other siderophore-based ligands that are structurally distinct from DFO [112].

**Figure 10:**
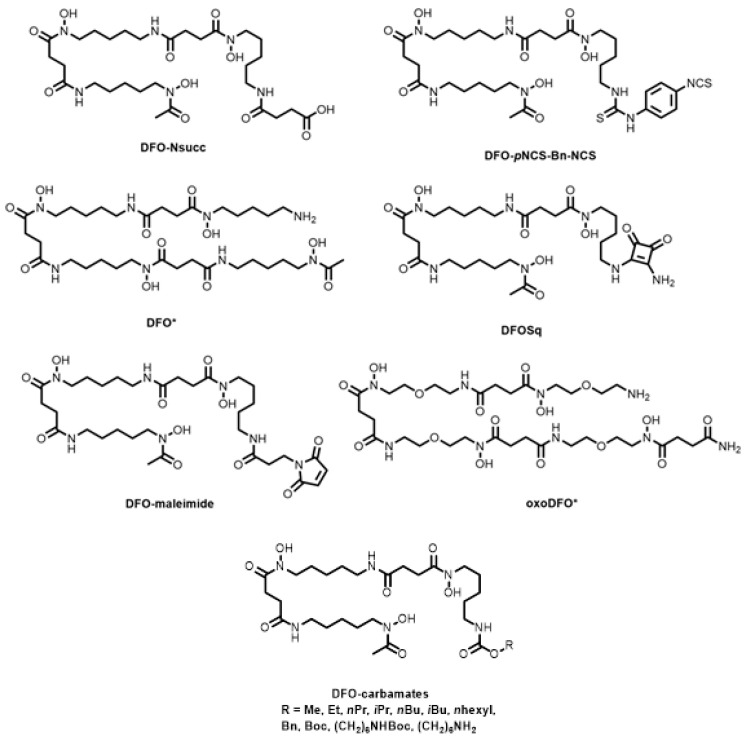
Bifunctional chelators and conjugates based on desferrioxamine-B.

Gourni and co-workers reported succinic acid (DFO-Nsucc, Figure 10) and isothiocyanate (DFO-*p*NCS-Bn-NCS, Figure 10) derivatives of DFO and subsequent conjugation to PSMA for ^68^Ga preclinical imaging of prostate cancer in mice bearing subcutaneous LNCaP tumours [114]. Compared to HBED-CC-PSMA, however, both conjugates showed decreased tumour uptake. Ioppolo and co-workers produced a library of alkyl-substituted DFO carbamates (-Me, -Et, -*n*Pr, -*i*Pr, -*n*Bu, -*i*Bu, -*n*hexyl, -boc, -Bn, (CH_2_)_6_NHboc and (CH_2_)_6_NH_2_, Figure 10) to tune the uptake of the ^67^Ga-labelled complexes in bacteria (*Staphylococcus aureus*) and sites of bacterial infection [115].

Octadentate derivatives of DFO (namely DFO *, Figure 10) were synthesised by incorporating a fourth hydroxamic acid and O atoms into the backbone structure for increased water solubility [116,117,118]. The resulting chelator, oxoDFO * (Figure 10), was more hydrophilic than DFO * and DFO, and was successfully radiolabelled with ^68^Ga at pH 4.5 and 95 °C, achieving >99% RCY.

The versatility of the DFO-squarate platform (DFOSq, Figure 10), where primary amine-containing compounds can be tethered together via the squaric acid group, was demonstrated with ^68^Ga radiolabelling and in vivo studies of octreotate, octreotide, and PSMA conjugates [119,120]. Similar derivatives of the free primary amine, including isothiocyanates and maleimides (DFO-maleimide, Figure 10), have been reported for the ^68^Ga PET imaging of tumour-induced angiogenesis and ^66^Ga radiolabelling of EGFR, respectively [121,122].

Bauman and co-workers and in a subsequent publication, Kaeppeli and co-workers, reported on DFO-Exendin 4 conjugates (using the bifunctional chelator, DFO-*p*NCS-Bn-NCS, Figure 10) for the radiolabelling of insulinomas and compared the radiolabelling as well as in vitro and in vivo stability to NODAGA and DTPA analogues [123,124]. Both studies demonstrated comparable tumour uptake with DFO outperforming NODAGA in terms of target-to-kidney ratio. DFO-*p*NCS-Bn-NCS was also used to radiolabel a short-chain variable fragment (scFv) targeting the human epidermal growth factor 2 (HER2) receptor [125]. The radiolabelled DFO-scFv conjugate showed high accumulation in HER2-positive xenograft-bearing mice and was used to monitor changes in HER2 expression following anti-HER2 therapy.

**Figure 11:**
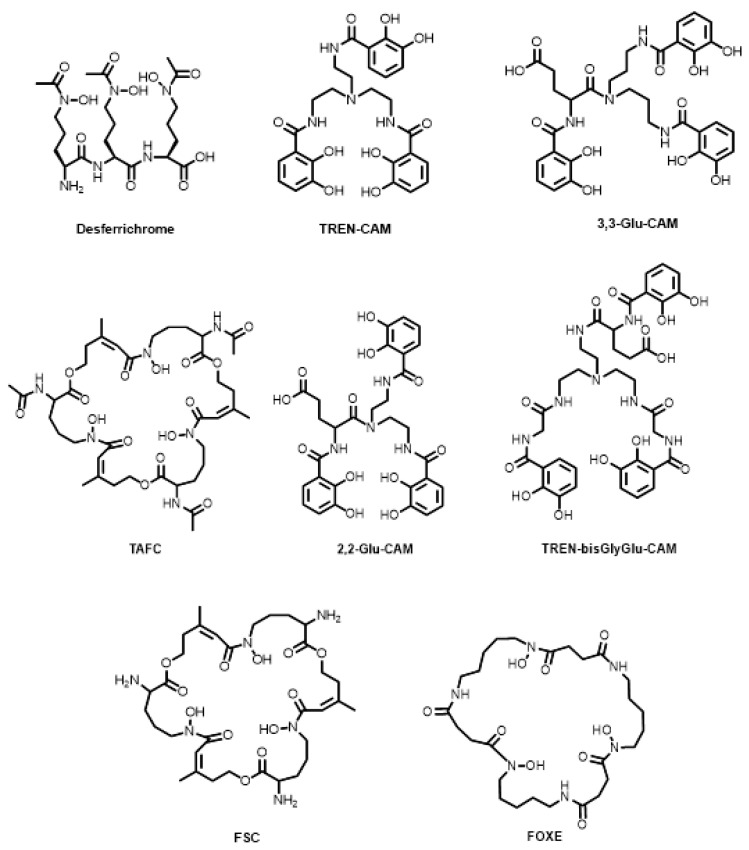
Bifunctional chelators and conjugates based on non-DFO siderophores.

Siderophores not based on the DFO scaffold have also seen developments over the last decade (Figure 11). Pandey and co-workers reported the conjugation of the desferrichrome chelator to the fluoroquinolone, ciprofloxacin, for ^68^Ga monitoring of a potential therapeutic for bacterial infection [105]. The catecholamide, enterobactin, is produced by the Enterobacteriaceae family of bacteria and has been shown to have one of the highest pFe values (35.5) [126] for all Fe^3+^ complexes (where the higher the pFe value, the more stable the complex), where pFe is defined as:

pFe = −log[Fe]_free_ when [enterobactin]_total_ = 10^−5^ M and [Fe]_total_ = 10^−6^ M

Joaqui-Joaqui and co-workers reported a class of enterobactin-based compounds, namely the catecholamides TREN-CAM, 2,2-Glu-CAM, 3,3-Glu-CAM, and TREN-bisGlyGly-CAM (Figure 11). The ^68^Ga complexes of TREN-CAM, 2,2-Glu-CAM, 3,3-Glu-CAM and TREN-bisGlyGly-CAM performed similarly to other acyclic ligands, including DFO, in terms of in vitro (human serum) and in vivo stability and in vivo renal clearance [108]. Petrik and co-workers reported the ^68^Ga radiolabelling of the siderophores, triacetylfusarinine C (TAFC, Figure 11) and ferrioxamine E (FOXE, Figure 11) for PET imaging of invasive pulmonary aspergillosis caused by the bacterium *Aspergillus fumigatus* [127]. TAFC was radiolabelled at room temperature for 15 min whereas FOXE required elevated temperature (80 °C) for 20 min for quantitative (>95% RCC) radiolabelling. Both hexadentate complexes were investigated in an in vitro model of *A*. *fumigatus* and showed rapid uptake in iron-deficient cultures. Follow-up studies were performed in vivo, showing vastly different organ uptake depending on the siderophore used, including DFO and fusarinine C (FSC, Figure 11) [128,129]. FSC is the deacetylated form of TAFC containing three primary amines that are separated from the hexadentate coordination sphere. Knetsch and co-workers followed by Zhai and co-workers demonstrated favourable ^68^Ga radiolabelling of FSC (>90% RCY after 5 min at room temperature) and conjugation to three RGD moieties through amide bond formation.

Given the similarity in coordination chemistry between Fe^3+^ and Ga^3+^, siderophores are a natural choice of chelator, and subsequent bifunctional chelator, for ^68^Ga radiopharmaceutical development. The resulting complexes are thermodynamically stable (e.g., log*K*_1_ ([Ga(DFO)] = 28.65) [130] but suffer from a lack of kinetic inertness, resulting in radiation leaching in vivo [108]. With the development of higher-denticity DFO ligands on the rise [116,117,118,131,132], these systems are seemingly better suited to other metallic radioisotopes, such as ^89^Zr^4+^. Exploring non-DFO siderophores may lead to better candidates for ^68^Ga radiolabelling, with the underexplored catechol- and catecholamide-based ligands a promising alternative.

## 8. Pyridinecarboxylate-Based Bifunctional Chelator Development

**Figure 12:**
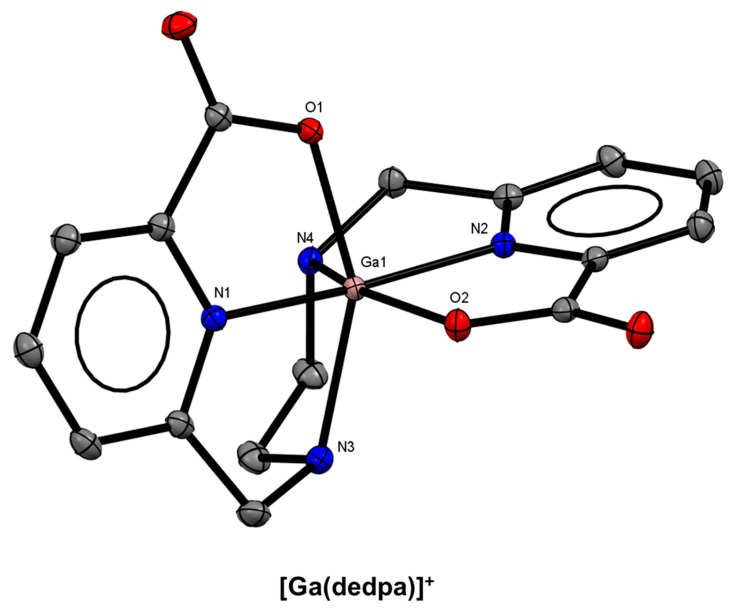
*ORTEP* representation of the [Ga(dedpa)]+ cation (CSD-OSUDOW) [133]. Ellipsoids are drawn at the 50% probability level. Hydrogen atoms, counterions, and solvent molecules are omitted for clarity.

**Figure 13:**
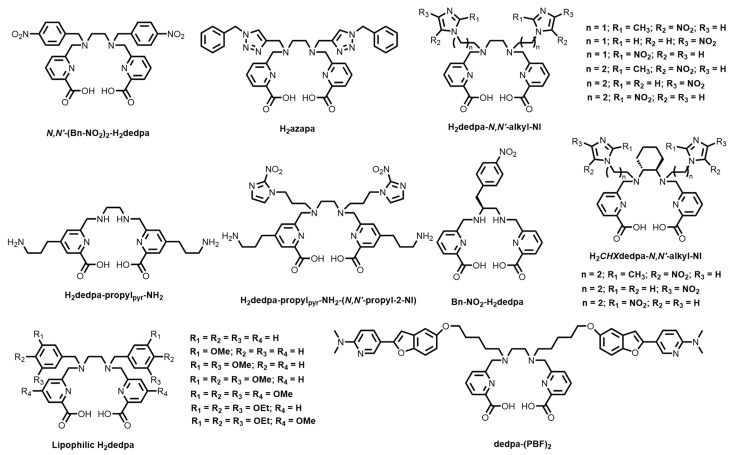
Bifunctional chelators based on the pyridinecarboxylate family of chelators.

In 2010, Boros and Orvig reported the synthesis, ^67/68^Ga radiolabelling, bioconjugation, stability, and biodistribution of an acyclic pyridinecarboxylate (“pa”) ligand, H_2_dedpa, based on earlier synthetic work by Platas-Iglesias and co-workers [133,134]. The X-ray crystal structure of the monocationic complex shows an N_2_O_4_, pseudo-octahedral coordination environment (Figure 12). The resulting ^68^Ga^3+^ complex (log *K*_ML_ = 28.11(8)) demonstrated quantitative radiolabelling at ligand concentrations as low as 10^−7^ M and has inspired the design and synthesis of several derivatives (Figure 13). Follow-up studies demonstrated the versatility of the bifunctional platform via conjugation to RGD peptides, synthesis of lipophilic analogues for myocardial imaging, as well as translation to copper-64 (^64^Cu) radiolabelling and subsequent in vitro and in vivo studies [135,136,137].

Bailey and co-workers reported a triazole dedpa derivative, H_2_azapa (Figure 13), that quantitatively radiolabelled ^67^Ga and ^64^Cu, as well as the large radiometal ions indium-111 (^111^In) and lutetium-177 (^177^Lu) [138]. Bis(propylamine) derivatives were also prepared (H_2_dedpa-propyl_pyr_-NH_2_ and H_2_dedpa-propyl_pyr_-NH_2_-(*N,N’*-propyl-2-NI, Figure 13) that show promise for bifunctionality via the reactive primary amine [139]. Bifunctional bi-modal fluorescent/nuclear imaging H_2_dedpa-fluorescein conjugates have also been reported, but showed that the bulky fluorescein moieties prevented effective ^67^Ga radiolabelling [140]. However, a recent report has shown that increasing the distance between the chelating unit (in this case, DOTA) and the fluorophore improves radiolabelling capabilities [89]. The analogous dedpa study has yet to be reported.

**Figure 14:**
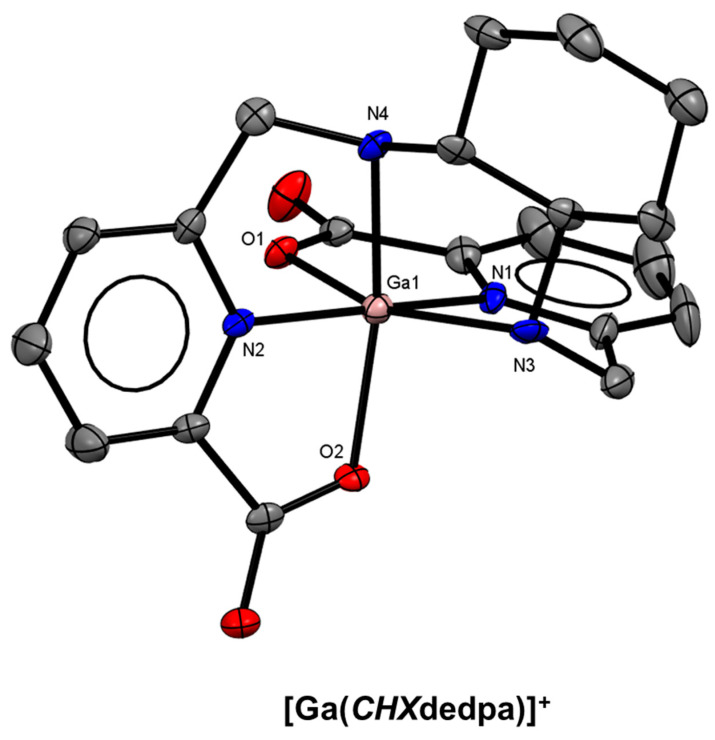
*ORTEP* representation of the [Ga(*CHX*dedpa)]^+^ cation (CSD-POXQOK) [42]. Ellipsoids are drawn at the 50% probability level. Hydrogen atoms, counterions, and solvent molecules are omitted for clarity.

Ramogida and co-workers investigated the effect of rigidifying the ethylenediamine backbone of H_2_dedpa by incorporating a chiral *trans*-cyclohexane ring. The resulting chelator, H_2_*CHX*dedpa (Figure 13) [42] was shown to form a more stable ^67^Ga^3+^ complex in human serum, and showed similar thermodynamic stability (log *K*_ML_ = 27.61(8)) and solid-state structure to H_2_dedpa (Figure 14). Nitroimidazole (NI) derivatives of both H_2_dedpa (H_2_dedpa-*N,N′*-alkyl-NI, Figure 13) and H_2_*CHX*dedpa (H_2_*CHX*dedpa-*N,N′*-alkyl-NI, Figure 13) were investigated for PET imaging of hypoxia using both ^68^Ga and ^64^Cu [139,140,141] Three H_2_dedpa-NI derivatives and one H_2_*CHX*dedpa-NI derivative were initially investigated in three in vitro cancerous cell line models (HT-29 (colon), LCC6^HER−2^ (breast), and CHO (ovary)) and all were found to have preferential uptake into hypoxic (0.5% O_2_) cells compared to normoxic (21% O_2_) cells, with hypoxic/normoxic ratios as high as 7.9 ± 2.7 after 2 h. Lipophilic analogues of H_2_dedpa containing a range of methoxy-substituted aromatic substituents (Figure 13) have also been reported with a view to develop heart imaging agents [142].

Saito and co-workers developed bifunctional H_2_dedpa derivatives bearing pyridylbenzofuran groups (dedpa-(PBF)_2_, Figure 13) for targeting islet amyloid deposition in pancreas islets, a key marker for Type 2 diabetes mellitus [143]. The authors were able to radiolabel the BFC with ^67/68^Ga but noted that improvement in non-target organ clearance was needed. Recently, Wang and co-workers reported 8-hydroxyquinoline (“hox”) analogues of both H_2_dedpa and H_2_*CHX*dedpa and investigated the preferential heart uptake of the Ga^3+^ complexes compared to the ‘pa’ family analogues [144,145]. Additionally, the inherent fluorescence of the ‘hox’ group shows promise for potential multi-modal PET/SPECT-fluorescence imaging in future.

Given the early promising results of the pyridinecarboxylate chelators, it is perhaps somewhat surprising that there have not been more pre-clinical and clinical studies reported. Moreover, the excellent kinetic inertness of the rigidified Ga^3+^ complexes make dedpa and its derivatives excellent choices for radiopharmaceutical development. Consequently, further exploration of their potential is warranted.

## 9. Hydroxypyridinone-Based Bifunctional Chelator Development

**Figure 15:**
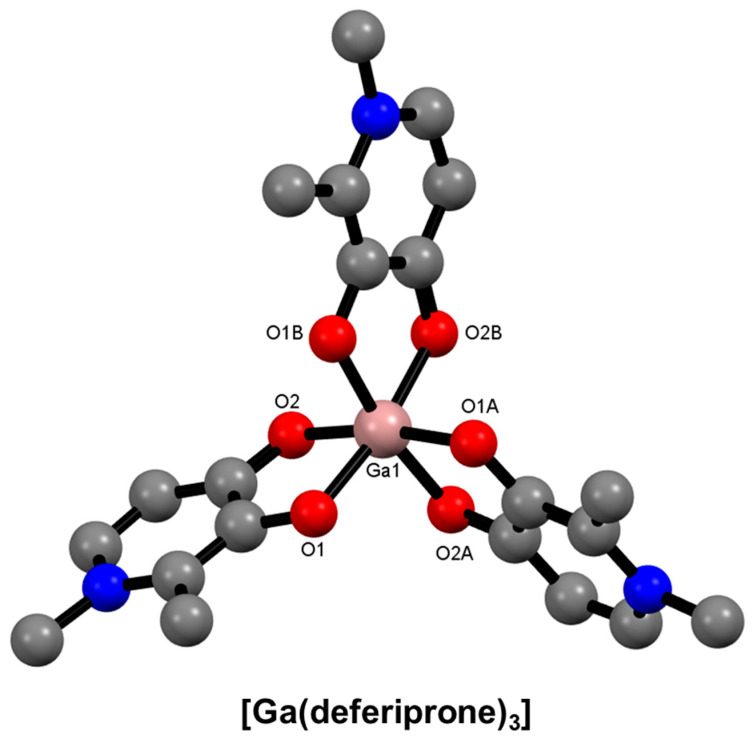
Representation of the molecular structure of [Ga(deferiprone)_3_] (*ORTEP* not reported) (CSD-GAVZIM) [151]. Hydrogen atoms and solvent molecules are omitted for clarity.

Hydroxypyridinones are a class of aromatic heterocycles that have been extensively studied for iron overload disease. They contain hydroxyl and ketone functional groups as donor atoms resulting in bidentate chelators [44,146,147]. Depending on the substitution pattern of the aromatic ring, three isomeric forms can exist: 1,2-hydroxypyridinones, 2,3-hydroxypyridinones, and 3,4-hydroxypyridinones. Binding to metal ions occurs with deprotonation of the acidic hydroxyl. The relative stability of the three Ga^3+^ complexes is known to follow the order 3,4-hydroxypyridinone > 2,3-hydroxypyridinone > 1,2-hydroxypyridinone, which reflects the charge density on the O atoms caused by the relative delocalisation of aromaticity around the ring [91,148,149,150]. 3,4-Hydroxypyridinones, such as deferiprone, form isostructural octahedral complexes with Fe^3+^ and Ga^3+^ (Figure 15).

Hexadentate tris(hydroxypyridinones) (THPs) have been of interest for Ga^3+^ complexation due to the 1:1 complexation stoichiometry compared to the 3:1 that would eventuate from tris(3,4-hydroxypyridinones). The first report of THP was in 2011 by Berry and Blower, where a radiolabelling study showed that THP could achieve higher RCY values than HBED, DOTA, and NOTA at pH 6.5 after 5 min at room temperature [146]. THPs have been synthesised from either tripodal polyamines or tripodal carboxylic acids, and dendritic constructs have also been reported [152].

**Figure 16:**
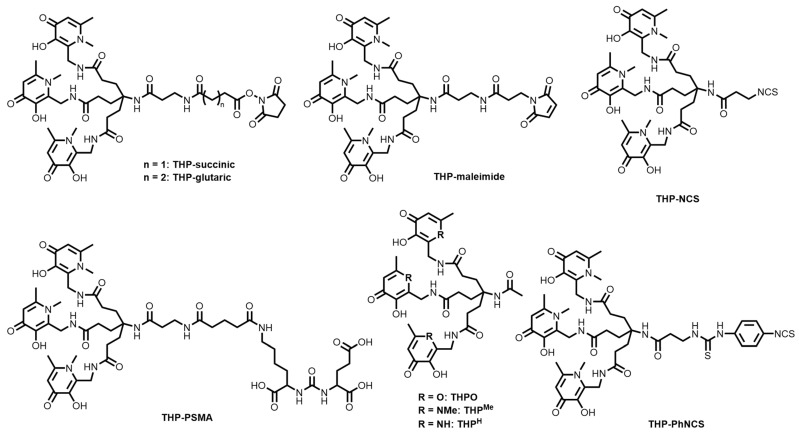
Bifunctional chelators and conjugates based on the hydroxypyridinone/THP family of chelators.

Over the past decade, several bifunctional THP derivatives have been reported (Figure 16) with the accompanying ^68^Ga^3+^ radiolabelling studies. Ma and co-workers reported the kit-based synthesis of [^68^Ga][Ga(THP-Tyr^3^-octreotate)] ([^68^Ga][Ga(THP-TATE)]) using an isothiocyanate derivative of THP (THP-NCS, Figure 16), and compared it against the FDA-approved [^68^Ga][Ga(DOTATATE)] in terms of somatostatin-2 receptor (SSTR2)-positive cell uptake [153]. THP-TATE was able to be radiolabelled within 2 min at >95% RCY (pH 5–6.5) and molar activity of 60–80 MBq/nmol, and proved comparable in vitro SSTR2-positive A427-7 cell uptake compared to [^68^Ga][Ga(DOTATATE)]. The biodistribution of [^68^Ga][Ga(THP-TATE)] in Balb/c nu/nu mice bearing SSTR2-positive AR42J tumours showed a clear delineation of the tumour as well as radiation uptake in the kidneys.

The conjugation of THP-NCS (Figure 16) and THP-PhNCS (Figure 16) to RGD peptides showed that both THP-peptide conjugates could be radiolabelled with ^68^Ga in under 5 min at >95% RCY at the same specific activity (60–80 MBq/nmol) as [^68^Ga][Ga(THP-TATE)] [154]. The radiolabelled conjugates showed uptake in αvβ3 integrin-positive glioblastoma U87MG tumours in Balb/c mice, and cleared from circulation within 2 h. However, both [^68^Ga][Ga(THP-TATE)] and the RGD conjugates have demonstrated lower tumour/non-target organ ratios compared to DOTA-based systems [154,155].

Imberti and co-workers explored dendritic variants of THP and synthesised three phenyl-isothiocyanate constructs conjugated to the RGD peptide (HP9-RGD3, HP3-RGD and HP3-RGD3) [152]. The HP9-RGD3 bioconjugate, containing three hexadentate metal binding sites, was shown to radiolabel at three times the specific activity value as the other two (180–240 MBq/nmol). However, it was demonstrated to have large uptake in non-target organs that compared unfavourably to the HP3-RGD3 construct. Although each of the chelators could quantitatively radiolabel ^68^Ga to 97% RCY, studies indicated that the distance between the RGD units was not large enough to sufficiently bind more than one integrin receptor.

Efforts have also been made towards synthesising THP bioconjugates for the PET imaging of prostate cancer. Nawaz and co-workers reported the conjugation of THP-maleimide (Figure 16) to a C-terminal cysteine residue of the scFv of the monoclonal antibody J591 that specifically binds to an external epitope of PSMA [156]. Radiolabelling of the THP-scFv conjugate proceeded at room temperature and neutral pH in a one-step synthesis, and the resulting radiotracer showed high affinity for PSMA in vitro and demonstrated uptake in a xenograft model (DU145-PSMA) of prostate cancer in mice. Blower and co-workers had arguably more success with their THP-PSMA conjugates, which can be radiolabelled in a kit-based, one-step synthesis at room temperature and near-neutral pH [29,157,158,159]. Phase I clinical trials have been reported and, in 2020, a 118-patient study was reported demonstrating the effectiveness of [^68^Ga][Ga(THP-PSMA)] in influencing clinical management of prostate cancer before therapy by identifying metastases in bone [159].

Despite the promising results with [^68^Ga][Ga(THP-PSMA)], the unfavourable comparisons of [^68^Ga][Ga(THP-TATE)] and the RGD conjugates with DOTA have motivated further tuning of the THP ligand design. Imberti and co-workers and Floresta and co-workers developed novel bifunctional THP derivatives incorporating changes to the hydroxypyridinone groups (THPMe, THPH, THPO, Figure 16) to tune the hydrogen-bonding and lipophilicity of the resulting ligands, as well as bifunctional pendant groups (THP-NHS, THP-succinic, THP-glutaric, THPMe-NCS, Figure 16) [147,155,160]. Preliminary radiolabelling studies have been reported, and work is ongoing to form radiolabelled bioconjugates with these promising systems.

The favourable radiolabelling properties of the THP chelators (near neutral pH and room temperature) are ideal for large biomolecules, such as proteins and antibodies [37]. Work is ongoing to translate THP and various bioconjugates to simple, rapid kit-based systems for clinical use, with the most promising to date being [^68^Ga][Ga(THP-PSMA)].

## 10. Diazepine-Based Bifunctional Chelator Development

**Figure 17:**
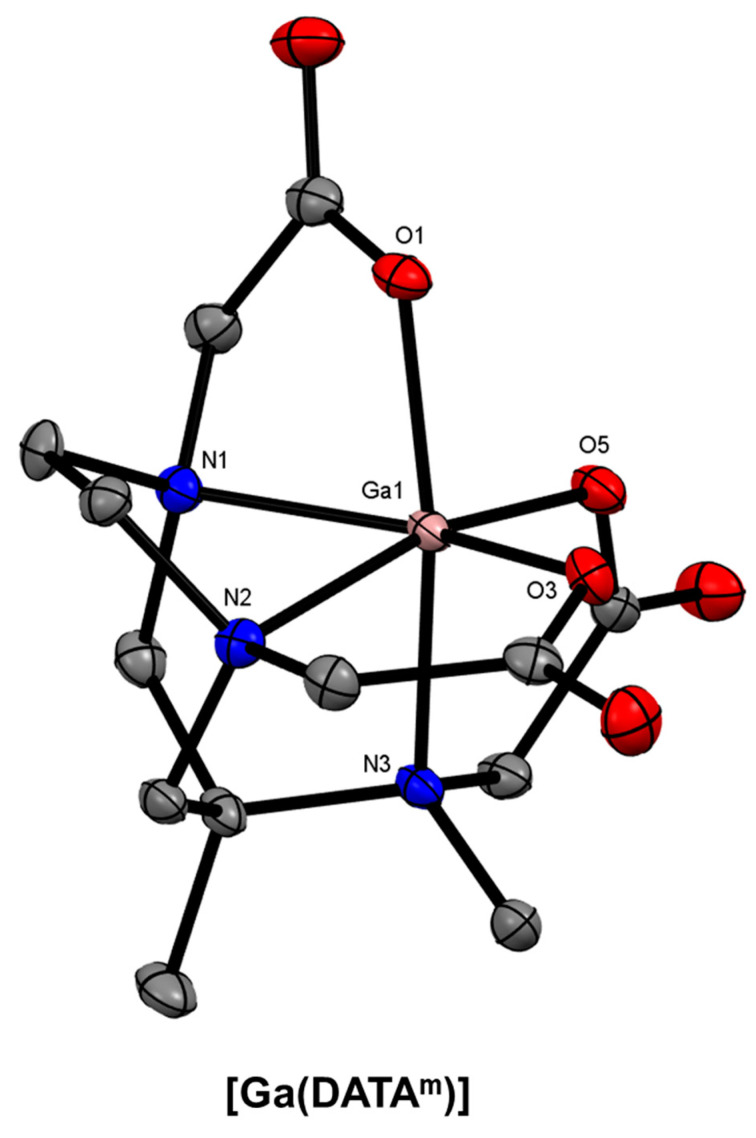
*ORTEP* representation of [Ga(DATA^m^)] (CSD-XENPAJ) [166]. Ellipsoids are drawn at the 50% probability level. Hydrogen atoms and solvent molecules are omitted for clarity.

The family of chelators based on the 6-amino-6-methyl-perhydro-1,4-diazepine (diazepine) scaffold has attracted much attention over the last two decades for ^68^Ga radiopharmaceutical development. This has been primarily spurred on by studies investigating the coordination chemistry of the parent and derivatised ligands between 2004 and 2009 (including transition metals and Gd^3+^ for MRI contrast agents), which highlighted the similarities between the resultant complexes and those of TACN, a constitutional isomer of diazepine [161,162,163,164,165]. The ligands form hexadentate Ga^3+^ complexes, generally in a facial (*fac*) arrangement of donor atoms with the N_3_ plane remaining consistent among the various complexes (Figure 17).

**Figure 18:**
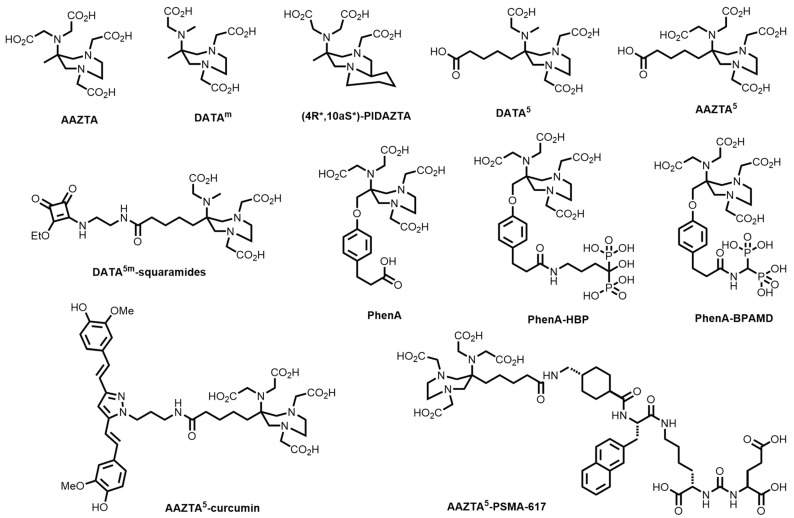
Bifunctional chelators and conjugates based on the diazepine family of chelators.

Waldron and Parker and co-workers reported three studies on the development of AAZTA (Figure 18) and various derivatives in 2013, showing hexadentate coordination of Ga^3+^, high kinetic inertness, and quantitative ^68^Ga radiolabelling at pH 4–7 [166,167,168,169]. Seeman and co-workers introduced the ‘DATA’ chelators, with the best performing, DATA^m^ (Figure 18), demonstrating 97% RCY in under 1 min at room temperature (23 °C) at a chelator concentration of 3.6 μM [43]. Farkas and co-workers then showed that the structurally constrained, bicyclic PIDAZTA (Figure 18) chelators coordinate ^68^Ga^3+^ at pH 7.5 (RCC > 89%) at concentrations as low as 10 μM [170].

Several groups have developed bifunctional variants of the diazepine chelators with various applications over the past decade. Wu and co-workers reported the synthesis of bisphosphonate AAZTA chelators (PhenA, PhenA-BPAMD, and PhenA-HBP, Figure 18) for the ^68^Ga PET imaging of bone [171]. The introduction of the bifunctional phenylcarboxylate pendant arm was enabled through tosylation of a methanol-diazepine derivative, originally reported in 2009 by Gugliotta and co-workers [172].

A pentanoic acid version of AAZTA, AAZTA^5^ (Figure 18) [173], was synthesised and conjugated to the cyclic peptide, Phe^1^-Tyr^3^-octreotide (TOC), via amide bond formation, and achieved quantitative radiolabelling (>98% RCC) at room temperature in less than 5 min at pH 4–5.5 at a ligand concentration of 10 nmol [174]. Hofstetter and co-workers tethered a Gastrin Releasing Peptide receptor (GRPr) antagonist, D-Phe-Gln-Trp-Ala-Val-Gly-His-Sta-Leu-NH_2_, to AAZTA^5^ via a 4-amino-1-carboxylmethyl-piperidine (Pip) spacer, for diagnostic imaging of GRPr-positive cancers (tested in an epithelial human prostate cancer cell line, PC3) [175]. Squaramide coupling has been utilised with both AAZTA^5^ and DATA^m^ (Figure 18) to conjugate to the fibroblast activation protein (FAP) inhibitor, UAMC-1110, and PSMA inhibitors [61,176,177,178]. DATA^5m^-squaramide (Figure 18) was tethered to the small molecule, UAMC-1110, and showed >97% RCY after 10 min and excellent stability in EtOH, HSA and saline over 2 h. An AAZTA^5^-squaramide was tethered to a Glu-urea-Lys motif for PSMA-targeting and radiolabelled successfully after 10 min at room temperature at pH 4–5.5.

AAZTA-curcumin conjugates (Figure 18) were reported by Orteca and co-workers for potential ^68^Ga PET imaging of colorectal cancer. The tetra-*tert*-butyl ester protected AAZTA was pre-activated with the coupling agent, HBTU, and reacted directly with curcumin to form the BFC [179]. Compared to the TACN derivative, NODAGA-curcumin, the AAZTA-curcumin conjugate showed greater stability in human blood over 2 h (with comparable stability in plasma and serum).

Yadav and co-workers were interested in comparing the biodistribution and gastroenteropancreatic neuroendocrine tumour (GEP-NET) uptake of [^68^Ga][Ga(DATA-TOC)], originally developed by Waldron and Parker, with [^68^Ga][Ga(DOTA-NOC)] (NOC = NaI^3^-octreotide), which required harsh (95 °C) radiolabelling conditions for quantitative coordination of ^68^Ga [180]. The authors showed that [^68^Ga][Ga(DATA-TOC)] demonstrated comparable PET/CT imaging of GEP-NET lesions (98.6% agreement with [^68^Ga][Ga(DOTA-NOC)], *n* = 235). This is promising, as DATA chelators can be more easily synthesised in a kit-based synthesis than DOTA-based systems [181,182].

The excellent radiolabelling conditions (very low ligand concentrations, neutral pH, and room temperature) make the diazepine family of chelators, particularly DATA^m^, a promising candidate for ^68^Ga radiopharmaceutical development. This facilitates preparation of instant kit-type preparations, which are crucial for successful clinical translation.

## 11. TACN-Based Bifunctional Chelator Development

Interest in TACN-based chelators has been spurred on by the particularly attractive coordination properties of triazamacrocyclic chelators with tricarboxylic acid (NOTA), triphosphonic acid (NOTP) and triphosphinic acid (TRAP) pendant groups for Ga^3+^. They are known to be highly rigid (due to the preformed geometry offered by the macrocycle), kinetically inert, and thermodynamically stable due in part to the macrocyclic effect (stability constants log*K_1_* > 26), which encompasses entropic gain from a pre-organised structure around the metal ions (Table 3) [52,183,184].

**Figure 19:**
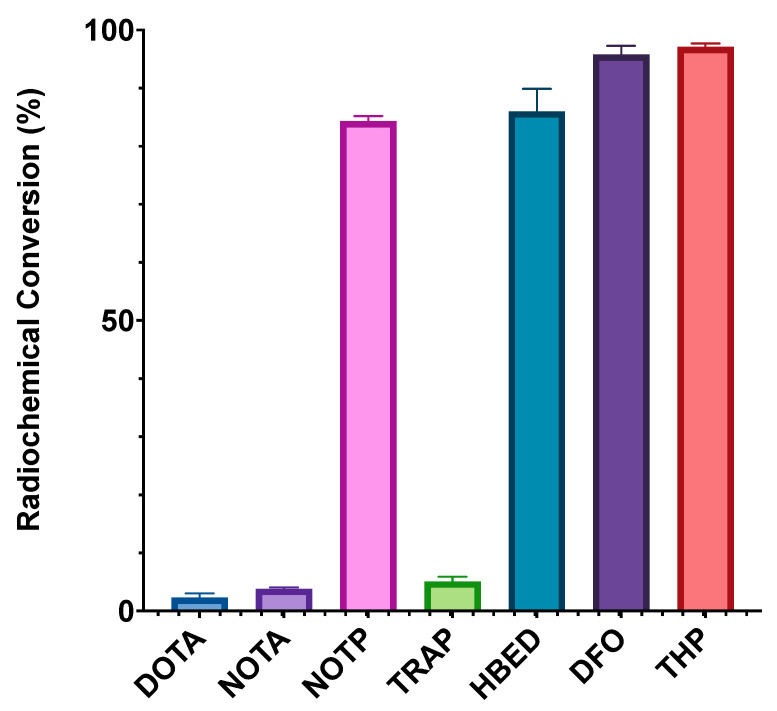
A comparison of the ^68^Ga radiolabelling efficiencies of THP, DFO, HBED, TRAP, NOTP, NOTA, and DOTA at pH 6.5, 25 °C after 10 min and a chelator concentration of 0.5 μM. Adapted from Ref. [38] with permission from the Royal Society of Chemistry [38].

In the case of [Ga(NOTA)], the X-ray crystal structure of the complex indicates that the distorted octahedral coordination environment contains three deprotonated carboxyl groups [189]. [Ga(TRAP)] exhibits similar coordination environments to [Ga(NOTA)], with deprotonated phosphinic acids contributing the O_3_ donor atoms. The radiolabelling properties of these chelators with ^68^Ga^3+^ have also been investigated. The triphosphinic acid, TRAP, was shown to radiolabel with ^68^Ga^3+^ to >95% RCC at a reduced ligand concentration (3 μM) compared to DOTA (500 μM) and NOTA (100 μM) at 25 °C and pH 0.5–5 [38,184]. Phosphonate-containing ligands such as NOTP have been shown to chelate ^68^Ga^3+^ at room temperature (25 °C) and neutral pH (6.5) at higher RCYs than DOTA, NOTA, and TRAP chelators at chelator concentrations of 0.5 μM (Figure 19) [38]. The X-ray crystal structure of [Ga(NOTP)] shows a hexadentate N_3_O_3_ coordination environment that is closer to ideal octahedral than [Ga(NOTA)] [190].

**Figure 20:**
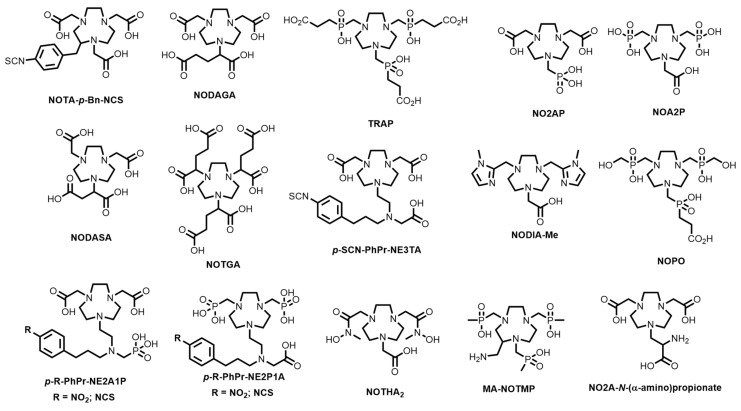
Bifunctional chelators based on TACN.

The simplest method of forming bifunctional variants of NOTA is through derivatisation of one carboxylic acid pendant arm (Figure 20). Coupling to an amine is achieved either through agents, such as HBTU or HATU or activated (sulfo)esters [191,192]. Less commonly used methods include the use of click compounds or thiol coupling [53,193,194]. However, these methods have a marked disadvantage, which is that the resulting amide is a worse coordinating group for Ga^3+^ than the carboxylic acid due to the lower thermodynamic stability of the resulting complexes [195,196].

**Figure 21:**
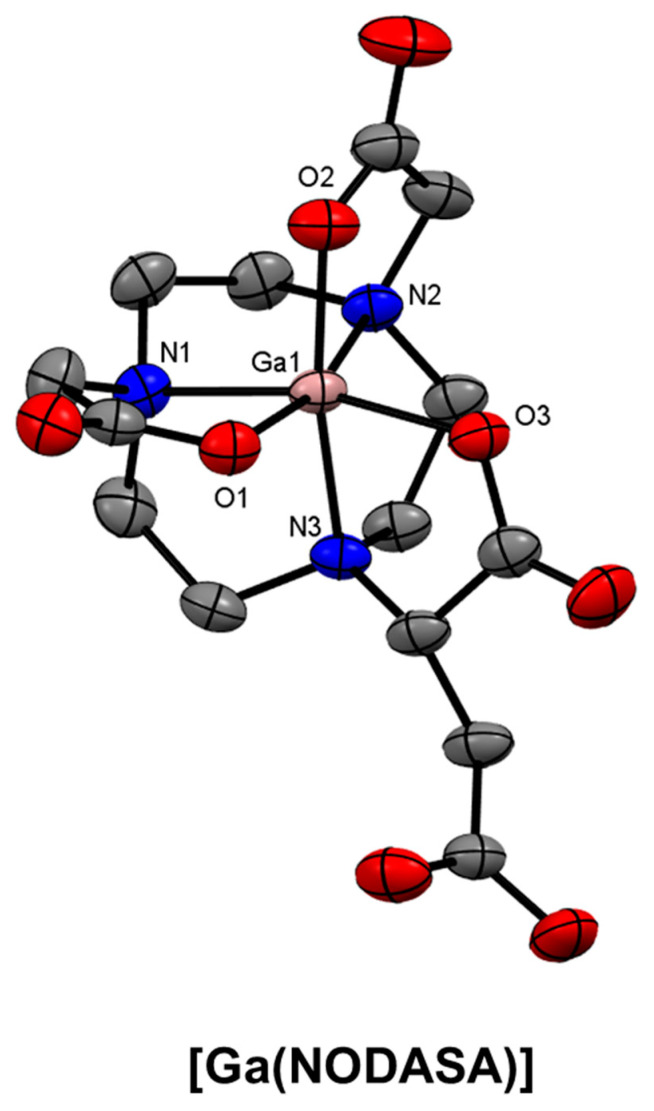
*ORTEP* representation of [Ga(NODASA)] (CSD-NUHLOR) [203]. Ellipsoids are drawn at the 50% probability level. Hydrogen atoms and solvent molecules are omitted for clarity.

Several well-known NOTA-based BFCs have been developed where an additional reactive functional group has been provided for conjugation reactions but does not interfere with the coordination environment. NODAGA (Figure 20), a glutaric acid analogue of NOTA, which was first reported in 2002 and has seen extensive conjugation and radiolabelling optimisation [55,56,62,124,197,198,199,200,201,202]. The succinic acid derivative, NODASA (Figure 20), was reported in 1998, but has seen less application than NODAGA, presumably due to their structural similarities and comparable radiolabelling efficiencies. The X-ray crystal structure of [Ga(NODASA)] shows a hexadentate N_3_O_3_ coordination environment that is typical of Ga^3+^ complexes of TACN and derivatives (Figure 21).

NOTA-p-Bn-NCS (Figure 20) is one of the most widely-studied BFCs for ^68^Ga, with a plethora of studies reporting its conjugation to targeting vector biomolecules, such as small molecules, peptides, and antibodies [100,204,205,206,207,208,209]. Massa and co-workers reported a triglycine derivative of NOTA-p-Bn-NCS, GGGYK-NOTA, for use in enzyme-mediated site-specific labelling of camelid single-domain antibody fragments with ^68^Ga [210]. Bone-targeting bisphosphonate-NOTA conjugates have been reported by Holub and co-workers and Passah and co-workers, demonstrating improved bone uptake compared to commercially available bone imaging agents [211,212]. Recently, the imidazole-based BFC, NODIA-Me (Figure 20), was reported and tethered to a PSMA targeting vector [213,214,215,216]. The resulting ^68^Ga complex and conjugate was kinetically inert to transmetallation in vivo, which underlined its potential use as a radiopharmaceutical agent.

Bifunctional variants of the phosphinate-containing TACN chelators, TRAP and NOPO (Figure 20), have also been synthesised and extensively evaluated [54,187,217,218,219,220,221,222,223,224]. which has been motivated by the ease by which the phosphinate pendant arms can be synthetically modified without affecting the Ga^3+^ coordination sphere O donor. Additionally, the distal carboxylic acids can be modified using uronium coupling agents (e.g., HATU) without protecting the phosphinic acids [189]. Dendritic TRAP ligands, containing four metal binding sites, have been prepared by tethering the macrocycles together via CuAAC click chemistry (through modification of the carboxylic acids with azides) [223].

TRAP was tethered to an α5β1 integrin-targeted trimeric pseudopeptide via modification of the carboxylic acids with three azides and subsequent CuAAC [218]. The TRAP-peptide conjugate, ^68^Ga-aquibeprin, showed high selectivity for integrin α5β1 (IC_50_ = 0.09 nM) over integrin αvβ3 (IC_50_ = 620 nM), and the ^68^Ga radiolabelled bioconjugate demonstrated a good tumour-to-blood ratio (10.6 ± 2.5, 90 min p.i.) in an ex-vivo biodistribution mice model xenografted with M21 human melanoma.

NOPO was evaluated as an αvβ3 integrin-targeting BFC via conjugation to the cyclic pentapeptide, c(RGDfK) [219]. The Ga^3+^ complex showed high affinity to αvβ3 integrin (IC_50_ = 1.02 nM) and showed a tumour-to-blood ratio of 19.6 ± 6.8 at 60 min p.i. in the same M21 mice model. A monomeric TRAP-azide construct has also been radiolabelled and tethered to an αvβ8 integrin targeting cyclic peptide [225].

TRAP-(azide)_3_ was tethered to Glu-urea-Lys for PSMA-targeting using CuAAC [224]. The trimeric conjugate displayed high PSMA affinity (IC_50_ up to 1.5 nM). Interestingly, lowering the molar activity of the radiolabelled conjugate from 1200 MBq/nmol to 8 MBq/nmol resulted in better kidney-to-tumour ratios (11.4 compared to 1.4, respectively) in subcutaneous murine PSMA-positive human prostate carcinoma xenografts. The trimethylphosphinate, MA-NOTMP (Figure 20), contains a reactive primary amine and has been used to tether the octapeptide, bombesin(7-14), which shows high affinity for three G protein-coupled receptors (overexpressed on a wide range of cancer types) [221]. The chelator was radiolabelled at RCC > 90% at pH 1.5–3 at 95 °C within 5 min but could not be radiolabelled >pH 4.

The presence of phosphonate pendant groups increases the thermodynamic stability and rigidity of the Ga^3+^ TACN chelates. For example, [Ga(NOA2P)] (Figure 20) has a high thermodynamic stability constant (log*K*_ML_ = 34.44) [226] compared to log*K*_ML_ = 29.63 for [Ga(NOTA)], and greater stability against apo-transferrin than [Ga(DOTA)] and [Ga(NOTA)] in competition experiments. Unlike the phosphinate-containing chelators, however, minimal bifunctional TACN chelators have been reported containing phosphonates [38]. This may be due to perceived difficulties with designing and synthesising BFCs. To date, the only BFCs that have been derivatives of NO2AP and NOA2P (Figure 20) were achieved by Gai and co-workers, who reported synthesis of bisphosphonate and monophosphonate-containing TACN BFCs (*p*-R-PhPr-NE2A1P and *p*-R-PhPr-NE2P1A, where R = NO_2_ or NCS, Figure 20) [183,226,227,228]. The development of novel bifunctional variants of phosphonate-containing TACN ligands is timely because, despite the excellent radiolabelling conditions displayed by [^68^Ga][Ga(NOTP)], [^68^Ga][Ga(NOA2P)] and [^68^Ga][Ga(NO2AP)], bifunctional analogues are sorely lacking [38].

## 12. Conclusions

Proper chelator design and an understanding of Ga^3+^ aqueous coordination chemistry are essential for the successful development of ^68^Ga radiopharmaceuticals. The key components of this development process include the use of well-designed chelators, capable of forming thermodynamically stable and kinetically inert ^68^Ga^3+^ complexes using mild conditions (near-neutral pH, room temperature and low concentrations of ligand) in a short timeframe (<10 min), which are tethered to vector biomolecules, such as peptides and antibodies.

The most promising ^68^Ga radiotracers are those that can be streamlined to pre-clinical and clinical applications using robust, reliable, and cost-efficient radiopharmaceutical kits. These include derivatives of THP, DATA, and TRAP, which have been applied to clinically relevant targeting vectors, such as RGD, octreotate, NaI^3^-octreotide, and PSMA-targeting motifs. Newer generation BFCs, such as PIDAZTA, non-DFO siderophores, and TACN phosphonates, fulfil the requirements for effective ^nat/67/68^Ga^3+^ complexation (N and O donor atoms, six-coordinate complexes, mild radiolabelling conditions), yet remain underexplored in terms of bifunctionality. Further developments in this area will continue to enable the effective and simple incorporation of ^68^Ga into new radiopharmaceuticals.

Finally, the choice of chelator has been shown to play a role in determining the biodistribution of small molecule and peptide-based radiopharmaceuticals. Therefore, assessing multiple BFCs to tune optimal biodistribution should be considered when utilising new targeting vectors.

## Figures and Tables

**Table 1:** Decay properties of medically relevant gallium radioisotopes.

Isotope	t_1/2_	Decay Mode	E (keV)	Production Method
^66^Ga ^67^Ga	9.5 h	β^+^ (56%)	β^+^, 4150, 935	Cyclotron, ^63^Cu(α,n)^66^Ga
		EC (44%)		
	78.2 h	EC (100%)	γ, 93, 184, 300	Cyclotron, ^68^Zn(p,2n)^67^Ga
^68^Ga	67.7 min	β^+^ (90%) EC (10 %)	β^+^, 1880	Cyclotron, ^68^Zn(p,n)^68^Ga; ^68^Ge/^68^Ga generator

**Table 2:** FDA-approved radiopharmaceuticals containing ^67/68^Ga [8].

Radiopharmaceutical	Manufacturer	Trade Name	Approved Indications in Adults
^67^Ga-gallium citrate	Curium; Lantheus Medical Imaging	-	Detection of lymphoma, bronchogenic carcinoma, Hodgkin’s disease.
[^68^Ga]Ga-DOTATATE	Advanced Accelerator Applications	NETSPOT^®^	Neuroendocrine tumours (adult and paediatric patients).
[^68^Ga]Ga-DOTATOC	University of Iowa	-	Gastroenteropancreatic tumours (adult and paediatric patients).
[^68^Ga]Ga-HBED-CC-PSMA	Advanced Accelerator Applications; Telix Pharmaceuticals Inc.; University of California	[^68^Ga]Ga-gozetotide; LOCAMETZ^®^; Illucix	PSMA-positive lesions in prostate cancer and associated metastases.

**Table 3:** Proton and Ga^3+^ affinity constants of example Ga^3+^ chelators. Adapted from Ref. [38] with permission from the Royal Society of Chemistry [38].

Chelator	log*K_a_*	log*K*_1_
NOTA	13.17, 5.74, 3.22, 1.96 [185]	29.63 [185]
NOTP	11.7, 9.1, 7.5, 5.8, 3.1, 0.9 [186]	-
TRAP	11.48, 5.44, 4.84, 4.23, 3.45, 1.66 [187]	26.24 [187]
DFO	10.79, 9.55, 8.96, 8.32 [130]	28.65 [130]
DOTA	11.74, 9.76, 4.68, 4.11, 2.37 [188]	26.05 [188]

## Data Availability

Not applicable.
